# Overexpression of PD-1 on Peripheral Blood Lymphocytes in Patients with Idiopathic Pulmonary Arterial Hypertension and Its Association with High Viral Loads of Epstein-Barr Virus and Poor Clinical Parameters

**DOI:** 10.3390/jcm9061966

**Published:** 2020-06-24

**Authors:** Michał Tomaszewski, Ewelina Grywalska, Andrzej Tomaszewski, Piotr Błaszczak, Marcin Kurzyna, Jacek Roliński, Grzegorz Kopeć

**Affiliations:** 1 Department of Cardiology, Medical University of Lublin, 20-954 Lublin, Poland; tomaszewskimd@gmail.com (M.T.); andrzej.tomaszewski@umlub.pl (A.T.); 2 Department of Clinical Immunology and Immunotherapy, Medical University of Lublin, 20-093 Lublin, Poland; jacek.rolinski@gmail.com; 3 Department of Cardiology, Cardinal Wyszynski Hospital, 20-718 Lublin, Poland; dr.piotr_blaszczak@wp.pl; 4 Department of Pulmonary Circulation, Thromboembolic Diseases and Cardiology, Centre of Postgraduate Medical Education, Fryderyk Chopin Hospital in European Health Centre Otwock, 05-400 Otwock, Poland; marcin.kurzyna@ecz-otwock.pl; 5 Department of Cardiac and Vascular Diseases, Faculty of Medicine, Jagiellonian University Medical College, Centre for Rare Cardiovascular Diseases, John Paul II Hospital, 31-202 Krakow, Poland; grzegorzkrakow1@gmail.com

**Keywords:** idiopathic pulmonary arterial hypertension, IPAH, cardiovascular disease, Epstein–Barr virus, EBV, PD-1, hypertension immunopathology

## Abstract

Idiopathic pulmonary arterial hypertension (IPAH) is a rare but severe disease with the elevated blood pressure in the pulmonary arteries without a known trigger of vascular remodelling. It leads to the right heart failure with reduced survival. Changes in the immunological landscape of the lungs and the periphery are common in IPAH patients, suggesting an immune system dysfunction. A cohort of 25 IPAH patients was enrolled in our study to investigate a link between the patient’s clinical status, immune parameters of the blood, and the Epstein–Barr virus (EBV) infection. We found significant alterations of the patients’ peripheral blood parameters. Therein, T lymphocytes and NK cell counts were decreased in the IPAH patients’ blood, while the proportion of regulatory T cells was increased. Additionally, levels of proinflammatory cytokines interleukin-6 (IL-6), IL-2, and interferon-gamma (IFN-γ) were elevated. We identified a weak correlation between EBV loads and IPAH patients’ clinical state (r = 0.54) and between EBV loads and overexpression of PD-1 on helper T cells (r = 0.56). We speculate that a significant dysregulation of the immune system homeostasis observed in IPAH patients may contribute to increased susceptibility of those patients to EBV infection, yet further longitudinal studies are required to characterize this relation in detail.

## 1. Introduction

Idiopathic pulmonary arterial hypertension (IPAH) is a sporadic disease that is associated with high morbidity and mortality rates. The disease affects the precapillary pulmonary vasculature and is characterised by a persistent hypertension in the pulmonary artery exceeding 25 mmHg, with a pulmonary artery wedge pressure (PAWP) ≤ 15 mmHg and pulmonary vascular resistance (PVR) > 3 Wood units (WU) [1]. The trigger of the disease is not defined, and when untreated, the disease may lead to the right ventricular dilatation resulting in right heart failure and eventually death of the patient [2]. Based on the symptoms severity and haemodynamic parameters, IPAH patients are classified into one of four WHO functional classes, with class I being the most benign and class IV comprising rapidly progressing patients with a high mortality risk and poor haemodynamic results [3].

Studies have shown that the immune landscape of patients suffering from IPAH is severely impacted, both in the lungs and in the periphery [4,5]. Significant changes in the cytokine profiles, such as IL-1β, IL-6, IL-8, MCP-1, RANTES, and TNF, were observed in IPAH patients when compared to healthy controls [5]. Indeed, some of the cytokines, e.g., IL-6, have been proposed as prognostic markers in assessing the clinical state and the survival of the patients [6]. It has also been observed that the cellular composition of the immune compartment can be altered in the IPAH patients. For example, lung tissue affected by IPAH has been shown to be infiltrated by CD4+ and CD8+ T lymphocytes [7,8], while the proportion of regulatory T cells (Treg) in the periphery was elevated [8]. Besides the cytokine markers, a significant deficiency in CD8+ T cells, as well as NK cells has been identified as a sign of deterioration of an IPAH patient’s status [9]. 

PD-1 (programmed cell death protein 1), known also as CD279, is an immune checkpoint protein expressed on the surface of T cells and certain B cells [10]. PD-1 on T cells serves as an inhibitor of the activating signals coming from TCR (T-cell receptor) and CD28, while PD-1-expressing B cells are regulatory toward T-cell responses [11,12]. Expression of PD-1 on T cells is induced during their activation, and PD-1 is abundantly present on the surface of activated and exhausted T cells [13]. PD-1 induces attenuation of the immune activation by interacting with two ligands: PD-L1 and PD-L2 (CD274 and CD273, respectively). PD-L1 is widely distributed in the body, being detected on T cells, B cells and antigen-presenting cells, as well as in non-immune tissues such as endothelium, placenta, and pancreatic islets [12]. PD-1/PD-L1 interaction may lead to inhibition of lymphocyte activation and proliferation and facilitates expansion of Treg. As a consequence, such an interaction limits tissue destruction that could occur during prolonged and uncontrolled inflammation, and thus, it protects the organism from auto-aggression [14,15]. 

Some viruses have been implicated in PAH development. Infection with human immunodeficiency virus (HIV) and chronic, active Epstein–Barr virus (EBV) infection are among the risk factors for developing PAH [16,17]. Moreover, a link between chronic, active EBV infection and development of PAH has been reported. Interestingly, the involvement of other viruses, such as human herpesvirus 8 (HHV-8), remains controversial. Some authors identified HHV-8 in samples obtained from PAH patients [18,19], while others found no link between HHV-8 infection and development of PAH [20,21]. 

In our study, we analysed blood samples obtained from 25 IPAH patients from various WHO functional classes of IPAH and 20 corresponding healthy controls. Haemodynamic parameters of the IPAH cohort, the EBV loads of patients and controls as well as their anti-EBV serological status were recorded. We noted that the IPAH patients were characterised by highly elevated levels of proinflammatory cytokines in the plasma. Proportions of CD4+ and CD8+ T cells, as well as NK cells in the blood, were decreased. At the same time, the number of Tregs was elevated, and PD-1 and PD-L1 were overexpressed on the surface of T and B lymphocytes. We found a weak but significant correlation between EBV copy number and the IPAH class and a link between EBV loads and expression of PD-1 molecules on CD4+ T cells. We have found that all EBV-positive IPAH patients had the antibodies indicating a past EBV infection; therefore, the observed positive EBV DNA test result suggests a viral reactivation. This outcome may suggest that the immune system dysregulation often observed during PAH may facilitate EBV reactivation, which may in turn exacerbate cardiovascular symptoms of IPAH patients.

## 2. Materials and Methods

### 2.1. Patients and Controls

We included 25 treatment-naïve, newly diagnosed patients with IPAH (10 men and 15 women) and 20 healthy volunteers (8 men and 12 women) matched for sex and age, who served as a control group. IPAH was diagnosed according to the criteria of the European Society of Cardiology and the European Respiratory Society [3]. The patients were recruited with the following inclusion criteria: no immunomodulatory treatment; no signs of infection at least three months before enrolment; no blood transfusion; no history of autoimmune, neoplastic, or allergic diseases. In patients with pulmonary hypertension, the functional class of heart failure was determined according to WHO criteria [3]. Each patient underwent complete blood count and natriuretic peptide (BNP) concentration assessments and a six-minute walk test. Basic laboratory tests were performed in the Alab laboratory of the Independent Public Clinical Hospital in Lublin. An echocardiographic examination of the heart (ECHO) was performed using a Phillips iE33 instrument. Cardiac catheterisation was carried out in the Haemodynamics Laboratory of the Independent Public Clinical Hospital and the Cardiology Department of the Provincial Specialist Hospital in Lublin, according to haemodynamic and angiographic assessment standards recommended by the Polish Cardiac Society [22]. The study was approved by the Ethics Committee of the Medical University of Lublin (KE-0254/309/2016)), and written informed consent was obtained from all participants. This study was conducted in accordance with the Helsinki Declaration. 

### 2.2. Preparation of Material

We collected 15 mL of peripheral blood in EDTA-coated tubes (Sarstedt, Nümbrecht, Germany). Blood from the EDTA-coated tubes was used to isolate peripheral blood mononuclear cells (PBMC) and plasma by density gradient centrifugation at 400 g for 30 min, no brake. Briefly, 5 mL of the whole blood diluted with 5 mL of saline was applied on 5 mL of Ficoll-Paque™ (Milteny Biotec, Bergisch-Gladbach, Germany). The PBMC layer was harvested; the cells were counted, and their viability was checked with trypan blue (0.4% trypan blue solution; Sigma Aldrich, Hamburg, Germany). Only PBMC samples with viability ≥95% were used. Plasma from the tubes with EDTA anticoagulant was stored at −80 °C until assayed.

### 2.3. Immunophenotyping

Immunophenotyping was performed using flow cytometry as previously described [23,24]. Cells were stained with a combination of monoclonal antibodies conjugated with different fluorochromes to determine the proportion of the specific cell types and the expression of PD-1 and PD-L1 on their surface. Percentages of PD-1-positive and PD-L1-positive T and B lymphocytes were measured using combinations of the following monoclonal antibodies: CD45/FITC, CD14/PE, CD3/CyChrome, CD19/FITC, CD4/FITC, CD8/FITC, CD279 (PD-1)/PE, and CD274 (PD-L1)/PE. Percentages of NK and natural killer T-like (NKT-like) cells were identified using CD3/FITC, CD16CD56/PE, and CD45/PerCP monoclonal antibodies, which allowed for the simultaneous assessment of CD3+ T lymphocytes and NK (CD16+CD56+) cells. CD19 expression was used as B cell marker. During analysis, the CD3+CD16+CD56+ (NKT-like cell) population was also determined. Monoclonal antibodies were purchased from BD Biosciences, USA. Additionally, the number of CD4+ CD25high FOXP3+ Treg cells in the CD4+ T lymphocyte subpopulation was determined using the Human Treg Flow kit (FOXP3 Alexa Fluor 488/CD4 PE-Cy5/CD25 PE, BioLegend, USA). The purity of the lymphocyte gate was evaluated by examining the distribution of cells in the coordinates of CD45 and CD14. The percentage of positive cells was established based on the signal detected in samples stained with isotype-matched, non-specific control antibodies. Three-colour immunofluorescence analyses were performed using a FACSCalibur flow cytometer (BD Biosciences, USA) equipped with a 488 nm argon laser. A minimum of 10,000 events were acquired and analysed using CellQuest Software (BD Biosciences, San Jose, CA USA). The percentages of cells expressing specific surface markers were calculated. Background fluorescence was determined, and the samples were gated to remove the cellular debris and include only single cells. 

### 2.4. Cytokine Quantification in the Plasma

Levels of IFN-γ, IL-6, IL-2, and IL-10 were determined in the plasma samples of IPAH patients and of the control group. Cytokine concentrations were measured using commercially available Quantikine ELISA kits from R&D Systems, Minneapolis, MN, USA. Human IFN-γ (sensitivity <8 pg/mL), human IL-2 (sensitivity <7 pg/mL), human IL-6 (sensitivity 0.03–0.22 pg/mL), and human IL-10 (sensitivity 0.03–0.17 pg/mL) were all purchased. The concentrations of cytokines were calculated based on standard curves and presented in pg/mL. All ELISA assays were performed according to the manufacturers’ instructions.

### 2.5. DNA Isolation and Calculation of EBV Load and Assessment of Anti-EBV Antibody Status

EBV loads were assessed as previously described [24]. DNA was isolated from 5 million PBMC with the QIAamp DNA Blood Mini Kit (QIAGEN, Hilden, Germany) according to the manufacturer’s instructions. The number of EBV-specific DNA copies in PBMC was calculated with the ISEX variant of the EBV polymerase chain reaction (PCR) kit (GeneProof, Brno, Czech Republic). A specific conservative DNA sequence for the EBV nuclear antigen 1 (EBNA-1) gene was amplified with real-time PCR. The number of viral DNA copies per μL of eluent was adjusted for the efficiency of DNA isolation, to be expressed as the viral DNA copy number per μg of DNA. All samples were examined in duplicate. A corresponding negative control (DNA elution buffer) was also included. The concentration and purity of the isolated DNA were verified with a BioSpec-nano spectrophotometer (Shimadzu, Kyoto, Japan). Due to the detection threshold of 10 EBV DNA copies per μL, all samples below this threshold were considered EBV negative (EBV(–)). PCR was performed with a 7300 Real Time PCR System (Applied Biosystems, Foster City, CA, USA). 

Additionally, anti-EBV serological status of the patients and the controls was assessed. EBV-specific antibodies (Ab) were detected using commercial enzyme-linked immunosorbent assays (ELISA; IBL International, Hamburg, Germany). IgA, IgM, and IgG antibody classes recognizing the early antigen (EA), viral capsid antigen (VCA), and EBNA-1 have been measured. The specific signals were obtained using ELISA Reader Victor TM3 (PerkinElmer, Waltham, MA, USA). Manufacturer-specified cut-offs have been applied.

### 2.6. Patients’ Infection Status Assessment

All samples were tested individually for the presence of the common bacterial, viral, and fungal pathogens. Bacterial (aerobic and non-aerobic) and fungal cultures were carried out under standard conditions, and they yielded no pathogens [24]. The presence of the genetic material of hepatitis C virus (HCV), hepatitis B virus (HBV), HIV, *Herpes simplex* virus 1 and 2 (HSV-1 and -2), cytomegalovirus (CMV), human papillomavirus (HPV), parvovirus B19, influenza virus, *Borrelia burgdorferi, Chlamydia trachomatis, Chlamydia pneumoniae, Mycobacterium tuberculosis, Toxoplasma gondii, Ureaplasma spp.,* and *Listeria spp*. was determined with PCR-based tests, and none of the samples gave a positive result [25,26]. Additionally, serological tests were performed for each patient and for each pathogen separately: HCV, HBV, HIV, HSV-1 and -2, CMV, HPV, parvovirus B19, influenza virus, *Borrelia burgdorferi, Chlamydia trachomatis, Chlamydia pneumoniae, Mycobacterium tuberculosis, Toxoplasma gondii, Ureaplasma* spp. and *Listeria* spp.

### 2.7. Statistical Analysis

Statistical significance was determined with a nonparametric Mann–Whitney test, and the *p* values below 0.05 were considered significant. Correlations between EBV loads and several different variables were assessed with Spearman’s rank test. *p* < 0.05 was considered significant. All calculations were completed using GraphPad Prism 7 software (GraphPad Software, La Jolla, CA, USA).

## 3. Results

### 3.1. IPAH Patients Have a More Immunosuppressive Blood Cell Profile than Healthy Controls

**Figure 1:**
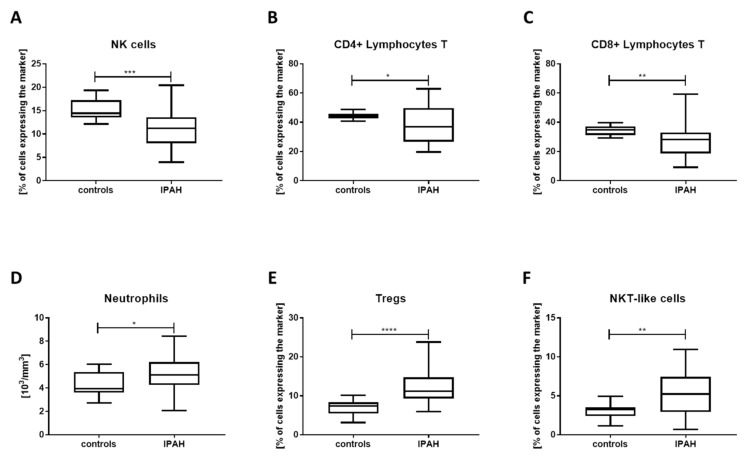
Idiopathic pulmonary arterial hypertension (IPAH) patients display altered cellular composition of the peripheral blood. Peripheral blood cells were immunophenotyped using flow cytometry, and specific cell populations were quantified. Data are presented as percentage of total leukocytes (CD45+ cells) or the absolute cell count in the blood for neutrophils. Graphs show comparison of IPAH patients’ results and corresponding healthy controls. There is a significant decrease in the proportions of (**A**) NK cells, as well as (**B**) CD4+ and (**C**) CD8+ T cells in the patients, as compared to controls. Moreover, ratios of (**D**) neutrophils, (**E**) T regs, and (**F**) NKT-like cells are augmented. Horizontal bars represent medians; boxes overlap 25th to 75th percentiles, and whiskers extend from minimum to maximum. In the figure, * denotes *p* ≤ 0.05, ** *p* ≤ 0.01, *** *p* ≤ 0.001, and **** *p* ≤ 0.0001.

PBMC isolated from patients suffering from IPAH were immunophenotyped, and the results were compared to a corresponding age- and sex-matched patient group (age: IPAH: min 23, median 62, max 81; controls: min 39, median 56, max 77; 60% of females in both groups). Whilst there were no significant changes in the fractions of total lymphocytes, B cells, and T cells (Table 1), we found that several immune cell populations, including NK cells, CD4+, and CD8+ T lymphocytes were significantly decreased in the blood of IPAH patients (Figure 1A–C, respectively). Levels of neutrophils were slightly elevated in the IPAH group (Figure 1D), while proportions of Treg cells (Figure 1E) and NKT-like cells (Figure 1F) were profoundly increased as compared to the controls. 

**Figure 2:**
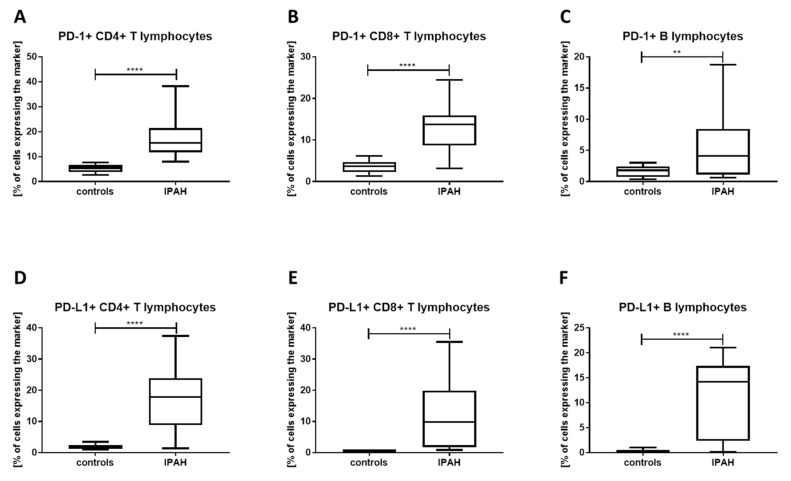
PD-1 and PD-L1 are overexpressed on T and B cells of IPAH patients. Expression of PD-1 and PD-L1 was assessed on B cells, CD4+, and CD8+ T cells. CD4+ T cells, CD8+ T cells, and B cells expressed (**A**–**C**) PD-1 and (**D**–**F**) PD-L1 markers at the significantly elevated levels in the IPAH patients’ blood. Horizontal bars represent medians; boxes overlap 25th to 75th percentiles, and whiskers extend from minimum to maximum. In the figure, ** denotes *p* ≤ 0.01 and **** *p* ≤ 0.0001.

Furthermore, percentages of T lymphocytes (both CD4+ and CD8+), as well as B cells expressing PD-1 (Figure 2A–C) and PD-L1 (Figure 2D–F), were highly elevated in IPAH patients. A summary of the blood cell immunophenotyping is presented in Table 1.

### 3.2. Proinflammatory Cytokine Levels in IPAH Patients’ Plasma are Elevated

**Figure 3:**
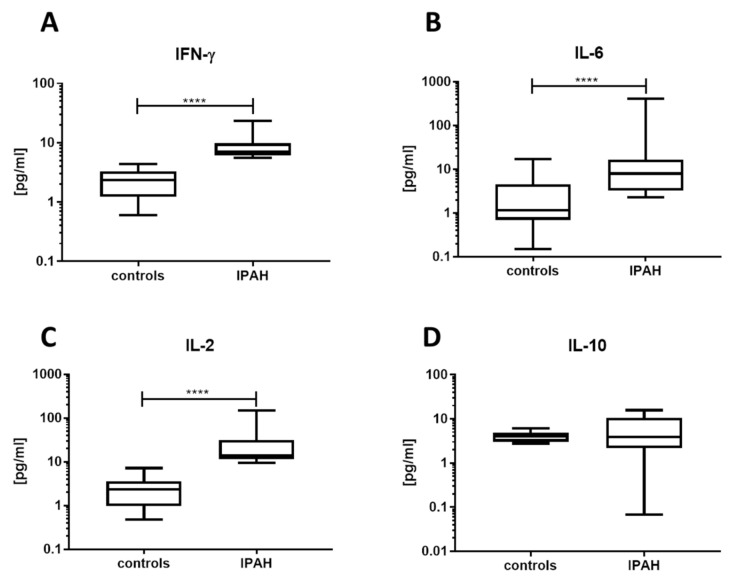
Levels of proinflammatory cytokines are increased in the IPAH patients’ plasma, while concentration of IL-10 remains unchanged. Plasma from IPAH patients and from healthy controls was assayed for (**A**) IFN-γ, (**B**) IL-6, (**C**) IL-2, and (**D**) IL-10. Levels of IFN-γ, IL-6, and IL-2 were highly increased in IPAH patients as compared to controls, and IL-10 was not statistically different between those two groups. Horizontal bars represent medians; boxes overlap 25th to 75th percentiles, and whiskers extend from minimum to maximum. Herein, **** denotes *p* ≤ 0.0001.

Concentrations of several cytokines were measured in the plasma of the IPAH patients and of the control group (Table 1). Here, patients suffering from IPAH had significantly increased levels of pro-inflammatory cytokines: IFN-γ, IL-6, and IL-2 (Figure 3A–C, respectively). Interestingly, levels of IL-10, an anti-inflammatory cytokine, were not significantly different between the patients and healthy controls (Figure 3D).

### 3.3. Haemodynamic Parameters of the IPAH Patients

IPAH patients were subjected to a series of tests assessing their haemodynamic parameters. The patients were classified into one of four WHO functional classes, from class I—comprising the least affected patients, to class IV—with the patients in the most severe condition [2]. There was one patient belonging to class I (4%), nine patients from class II (36%), 12 patients from class III (48%), and three patients presented characteristics of class IV (12%). The details of the haemodynamic parameters are presented in Table 2.

### 3.4. EBV Status is Correlated with the Severity of Clinical Manifestation of IPAH and Expression of PD-1 on CD4+ T Cells

**Figure 4:**
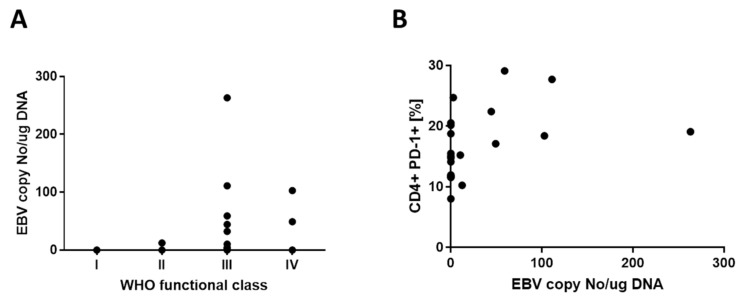
Positive correlation between WHO functional class and the EBV copy number and EBV load and the proportion of PD-1-expressing T helper lymphocytes. Weak, yet statistically significant correlations have been observed for (**A**) WHO functional class and the EBV copy number (r = 0.54, *p* = 0.005) as well as for (**B**) and EBV load and the proportion of PD-1-expressing CD4+ T cells (r = 0.56, *p* = 0.0035). Correlation was assessed with the Spearman rank test, and no regression line is plotted on the graphs.

Blood cells from IPAH patients and healthy controls were subjected to EBV load quantification. Importantly, none of the patients nor the controls had a positive result with regard to common pathogen infection, including hepatitis C virus (HCV), hepatitis B virus (HBV), HIV, *Herpes simplex* virus 1 and 2 (HSV-1 and -2), cytomegalovirus (CMV), human papillomavirus (HPV), parvovirus B19, influenza virus, *Borrelia burgdorferi, Chlamydia trachomatis, Chlamydia pneumoniae, Mycobacterium tuberculosis, Toxoplasma gondii, Ureaplasma* spp., and *Listeria* spp. All samples from the healthy volunteers were negative for EBV, while forty-four per cent (11 out of 25) of all IPAH patients were positive in the EBV test. The viral load of the EBV-positive patients varied from 2.71 to 263.14, with the median of 44.34 EBV copy numbers per 1 μg of DNA. Analysis of correlation performed with the Spearman test revealed that the EBV copy number was positively correlated with the severity of IPAH (WHO functional class), r = 0.54, *p* = 0.005 (Figure 4A). Furthermore, a positive correlation was also found for EBV load and the proportion of PD-1-expressing T helper lymphocytes: r = 0.56, *p* = 0.0035 (Figure 4B). 

Interestingly, analysis of the serological status of the study participants revealed that all IPAH patients with positive EBV DNA results had antibody titres indicating a viral reactivation, assessed as described by De Paschale et al. [27] Samples of all of them were positive for anti-VCA IgM, anti-VCA IgG, and anti-EBNA-1 IgG, while results of anti-EA IgM and anti-EBNA-1 IgM were negative. Additionally, four patients had anti-EBNA-1 IgA. On the other hand, IPAH patients with negative results of the EBV DNA test as well as all healthy controls had the anti-EBV status suggesting a past EBV infection (anti-VCA IgM negative; anti-VCA IgG positive; anti-EBNA-1 IgG positive; anti-EA IgM, IgG, and IgA negative; anti-VCA IgA negative; and anti-EBNA-1 IgA and IgM negative).

## 4. Discussion

Development of PAH has been observed in patients with immunological disturbances such as systemic sclerosis and chronically infected people such as those infected with HIV [28,29]. This suggests that a prolonged, dysregulated inflammatory response may contribute to the pulmonary arterial remodelling by increased vascular smooth muscle density, which is a hallmark of PAH [28]. In our study, we found a weak but statistically significant correlation between the EBV load and the severity of the clinical status of IPAH patients determined according to the WHO functional classification. Furthermore, we also identified a correlation between the expression of PD-1 protein on CD4+ T lymphocytes and the EBV load. We found that in comparison with healthy controls, IPAH patients had decreased levels of CD4+ and CD8+ T lymphocytes and NK cells in the blood. Instead, levels of Tregs, NKT-like cells and neutrophils were increased, and PD-1 and PD-L1 proteins were overexpressed on both T and B cells. 

It was reported previously that increased numbers of several immune cell types such as CD8+ cytotoxic T cells, CD4+ helper T cells, dendritic cells and macrophages were found in the lung tissue of IPAH patients [7,8]. Simultaneously, IPAH patients had more regulatory T cells in the periphery [8], and Ulrich et al. have additionally noted less cytotoxic T lymphocytes in the peripheral blood [30]. It is now well established that a significant immune dysregulation is associated with IPAH [4]. Furthermore, it has been suggested that a deficiency in NK and cytotoxic T cells might be linked with a poorer prognosis for IPAH patients, resulting in their increased risk of death [9]. Similarly, in our cohort, a significant decrease in the levels of NK cells, and CD8+ and CD4+ T cells was noted, which further supports the fact that IPAH patients display a deficiency in the cytotoxic immune cell compartment in the periphery. We also confirmed the observation of Austin et al. that the proportion of Treg cells is elevated in IPAH patients [8]. Additionally, we identified NKT-like cells, known as an important source of IFN-γ [31], as substantially upregulated in the blood of IPAH patients. 

Activation of lymphocytes during infection or due to autoimmune disorders results in overexpression of PD-1 protein on their surface [14,15,32]. In parallel, levels of PD-L1 are also increased, which allows for regulation of the immune response to limit its overactivation and to protect normal tissue function [15,32]. PD-1/PD-L1 interaction leads to inhibition of proliferation and impacts the cytokine release triggered by activation of TCR [12]. An increase in PD-1 expression is also linked with T-cell exhaustion, which is a T cell dysfunction characterized by altered proliferating abilities, a changed cytokine profile and a loss of T cell effector function [33]. On the other hand, it has been suggested that the PD-1/PD-L1 interaction may not necessarily lead to exhaustion but only indicates constant immune cell activation due to chronic infection with maintained capability of cells to be activated and respond to various stimuli [34]. In fact, Utzschneider et al. have shown that a chronic infection elicited T cells that were not functionally impaired and were able to transition into the memory compartment [35]. Importantly, upregulation of PD-L1 does not fully parallel PD-1 expression due to several factors, such as timing or cell type expressing PD-L1; therefore, a mere PD-L1 overexpression does not indicate the immune system homeostasis [35,36]. Overexpression of PD-1 and PD-L1 induced by EBV infection has been observed in various malignancies, including Hodgkin lymphoma and gastric cancers [37,38]. We found that patients enrolled in our study had increased levels of PD-1 and PD-L1 on CD4+ and CD8+ T cells, as well as on B cells. Moreover, we found that expression of PD-1 on CD4+ T cells correlated with EBV load. Additionally, the clinical status of IPAH patients, assessed according to the WHO functional classification, was also related to the EBV copy number, which suggests that people suffering from IPAH might have major problems maintaining immune system homeostasis and thus be more susceptible to EBV. Nonetheless, it remains to be elucidated whether overexpression of PD-1/PD-L1 molecules on the surface of lymphocytes in the blood of the IPAH patients is associated with their exhausted phenotype or that those cells remain fully functional. 

The role of various proinflammatory cytokines in the development and progression of PAH has been widely studied. Interestingly, Soon et al. reported that levels of IL-6, IL-8, and IL-12 predicted survival of patients and the level of IL-6 has been a better predictive marker than standard methods, such as six-minute walk distance and haemodynamic parameters [6]. Of note, in our study, plasma levels of IFN-γ, IL-6, and IL-2, cytokines related to the inflammatory state of the immune system, were highly elevated in the samples derived from IPAH patients as compared to healthy controls, whereas there was no correlation of the cytokine levels and EBV loads.

Some viruses may influence blood vessel remodelling, and development of atherosclerosis or hypertension [18,39]. Severe pulmonary hypertension is one of the cardiovascular complications of HIV infection [16]. Other viruses such as cytomegalovirus, EBV, or Herpes simplex type 1, as well as Helicobacter pylori and Mycoplasma pneumonia, have been found to contribute to development of atherosclerosis [39]. Moreover, the contribution of herpesviruses to development of PAH has been already examined. HHV-8, a vasculotropic virus, has been implicated by some authors in the development of primary pulmonary hypertension [18,19]. Other studies, however, do not confirm a link between HHV-8 infection and PAH [20,21,40]. Furthermore, EBV has been reported among factors contributing to PAH development [17,41,42] However, no studies have investigated a potential link between EBV infection and severity of the IPAH patients’ condition.

### Limitations of the Study

In our study, we included a carefully chosen group of newly diagnosed IPAH patients with strict inclusion criteria, such as no infection three months prior to the study, no immunomodulatory treatment, no allergy, etc. Since IPAH is a rare disease, we only found 25 patients fulfilling the criteria, and we deeply analysed samples obtained from those patients and matched them with other clinical values such as haemodynamic parameters. A larger study group could deliver more statistically significant correlations or differences between IPAH patients and healthy controls. Additionally, longitudinal studies on how the WHO functional class of the study participants would change over time and whether the progression towards higher classes would correlate with the EBV loads in the patients would deliver an important information. Indeed, we plan to follow the patients described in this study and add the new data on how and whether the IPAH status changes will be reflected by the changes in the EBV loads.

## 5. Conclusions

Our observations shed light on the impact of EBV on the clinical status of IPAH patients. We speculate that a viral reactivation may appear due to the immune system imbalance typically observed in IPAH patients. However, it requires further studies to establish whether infection with EBV may also predispose people to develop pulmonary arterial hypertension—as it is observed for HIV [16]. We rather hypothesise that the loss of immune homeostasis seen in IPAH patients contributes to a greater susceptibility of those people to EBV reactivation and possibly also de novo infection, which in turn leads to even more severe immune system dysregulation paralleled by aggravation of the IPAH symptoms. 

## Figures and Tables

**Table 1:** Results of the immunophenotyping of the peripheral blood and the plasma cytokine determination of the IPAH patients and corresponding controls. Cells were enumerated and presented either as No/vol or proportion of cells given as % of specific subpopulation within the leukocyte (CD45+) population. Statistical significance was determined with nonparametric Mann–Whitney test; p values below 0.05 were considered significant, and * denotes *p* ≤ 0.05, ** *p* ≤ 0.01, *** *p* ≤ 0.001, and **** *p* ≤ 0.0001.

Variable (Unit)	Group	Minimum	25th Percentile	Median	75th Percentile	Maximum	*p*
**Neutrophils (10^3^/mm^3^)**	Control	2.710	3.595	3.935	5.373	6.030	0.0378 (*)
	IPAH	2.080	4.250	5.110	6.225	8.430	
**Lymphocytes (10^3^/mm^3^)**	Control	1.530	1.988	2.535	2.785	3.070	NS
	IPAH	1.200	1.790	2.010	2.680	3.140	
**T lymphocytes (%)**	Control	60.63	65.52	68.08	70.93	74.49	NS
	IPAH	59.21	65.86	70.82	75.92	89.16	
**B lymphocytes (%)**	Control	6.04	9.86	11.40	12.37	16.90	NS
	IPAH	3.50	6.30	11.90	15.47	20.67	
**NK cells (%)**	Control	12.16	13.54	14.43	17.22	19.34	0.0002 (***)
	IPAH	3.99	8.05	11.23	13.57	20.43	
**NKT-like cells (%)**	Control	1.15	2.42	3.27	3.51	4.92	0.0043 (**)
	IPAH	0.67	2.92	5.23	7.45	10.94	
**CD4+** **T lymphocytes (%)**	Control	40.71	42.57	44.16	45.95	48.84	0.0190 (*)
	IPAH	19.73	26.69	36.91	49.78	62.92	
**CD8+** **T lymphocytes (%)**	Control	29.33	31.22	34.74	37.25	39.60	0.0034 (**)
	IPAH	9.19	18.73	28.30	33.01	59.29	
**CD4+/CD8+ ratio**	Control	1.030	1.188	1.290	1.433	1.570	NS
	IPAH	0.340	0.785	1.250	3.065	6.850	
**Treg (%)**	Control	3.15	5.49	7.37	8.33	10.15	<0.0001 (****)
	IPAH	5.94	9.31	11.21	14.73	23.81	
**PD1+ CD4+** **T lymphocytes (%)**	Control	2.65	3.83	5.35	6.64	7.69	<0.0001 (****)
	IPAH	8.00	11.73	15.49	21.46	38.24	
**PD-L1+ CD4+** **T lymphocytes (%)**	Control	0.98	1.30	1.71	2.52	3.49	<0.0001 (****)
	IPAH	1.43	8.83	17.84	23.91	37.42	
**PD-1+ CD8+** **T lymphocytes (%)**	Control	1.36	2.293	3.705	4.668	6.17	<0.0001 (****)
	IPAH	3.19	8.65	13.77	15.94	24.43	
**PD-L1+ CD8+** **T lymphocytes (%)**	Control	0.31	0.35	0.43	0.53	0.67	<0.0001 (****)
	IPAH	0.87	1.75	9.82	19.84	35.47	
**PD-1+** **B lymphocytes (%)**	Control	0.37	0.76	1.81	2.44	3.01	0.0011 (**)
	IPAH	0.62	1.12	4.13	8.45	18.76	
**PD-L1+** **B lymphocytes (%)**	Control	0.07	0.14	0.20	0.28	1.03	<0.0001 (****)
	IPAH	0.14	2.37	14.20	17.39	21.05	
**IFN-γ (pg/mL)**	Control	0.606	1.233	2.336	3.329	4.378	<0.0001 (****)
	IPAH	5.579	6.092	7.044	9.738	23.300	
**IL-6 (pg/mL)**	Control	0.151	0.694	1.168	4.567	17.200	<0.0001 (****)
	IPAH	2.304	3.255	7.959	16.790	411.400	
**IL-10 (pg/mL)**	Control	2.775	3.045	4.081	4.800	6.157	NS
	IPAH	0.068	2.144	3.908	10.570	15.710	
**IL-2 (pg/mL)**	Control	0.478	0.978	2.360	3.563	7.155	<0.0001 (****)
	IPAH	9.387	11.380	13.800	31.850	150.100	

**Table 2:** IPAH patient characteristics. Haemodynamic parameters, as well as BMI and levels of BNP of 25 IPAH patients are presented with the medians, minima, maxima, and 25th and 75th percentiles.

Parameter	Minimum	25th Percentile	Median	75th Percentile	Maximum
**BMI (kg/m^2^)**	17.1	24.1	26.0	30.6	40.5
**BNP (pg/mL)**	210	666	1546	2129	10,144
**6MWD (m)**	136	315	374	454	556
**PVR (mmHg x min-l)**	158	473	651	894	1599
**CI (l/min/m^2^)**	1.43	2.01	2.60	2.92	3.75
**CO (l/min)**	2.11	3.99	4.46	5.40	6.42
**RAP (mmHg)**	2	4	9	12	23
**MPAP (mmHg)**	25	32	48	53	66
**PASP (mmHg)**	37	55	77	89	105
**RVSP (mmHg)**	42	52	76	82	96
**PAWP (mmHg)**	5	7	9	10	14

BMI—body mass index, BNP—B-type natriuretic peptide, 6MWD—6-min walking distance, PVR—pulmonary vascular resistance, CI—cardiac index, CO—cardiac output, RAP—right atrial pressure, MPAP—mean pulmonary artery pressure, PASP—pulmonary artery systolic pressure, RVSP—right ventricular systolic pressure, PAWP—pulmonary artery wedge pressure.
